# Development of the Malay Language of understanding, attitude, practice and health literacy questionnaire on COVID-19 (MUAPHQ C-19): content validity & face validity analysis

**DOI:** 10.1186/s12889-023-16044-5

**Published:** 2023-06-13

**Authors:** Izzaty Dalawi, Mohamad Rodi Isa, Xin Wee Chen, Zahir Izuan Azhar, Nazim Aimran

**Affiliations:** 1 Training Reserve Position Unit, Training Management Division, Ministry of Health Malaysia, Level 6, Prisma Tower, Precinct 3, Federal Territory of Putrajaya, 62675 Malaysia; 2 Department of Public Health Medicine, Faculty of Medicine, Universiti Teknologi MARA, Sungai Buloh Campus, Sungai Buloh, 47000 Selangor Malaysia; 3 School of Mathematical Sciences, College of Computing, Informatics and Media, Universiti Teknologi MARA, Shah Alam, 40450 Selangor Malaysia

**Keywords:** Content validity, COVID-19, Face Validity, KAP, Malay

## Abstract

**Objective:**

This study aimed to assess the content and face validity index of the development of the understanding, attitude, practice and health literacy questionnaire on COVID-19 (MUAPHQ C-19) in the Malay language.

**Methods:**

The development of the MUAPHQ C-19 was conducted in two stages. Stage I resulted in the generation of the instrument’s items (development), and stage II resulted in the performance of the instrument’s items (judgement and quantification). Six-panel experts related to the study field and ten general public participated to evaluate the validity of the MUAPHQ C-19. The content validity index (CVI), content validity ratio (CVR) and face validity index (FVI) were analysed using Microsoft Excel.

**Results:**

There were 54 items and four domains, namely the understanding, attitude, practice and health literacy towards COVID-19, identified in the MUAPHQ C-19 (Version 1.0). The scale-level CVI (S-CVI/Ave) for every domain was above 0.9, which is considered acceptable. The CVR for all items was above 0.7, except for one item in the health literacy domain. Ten items were revised to improve the item’s clarity, and two items were deleted due to the low CVR value and redundancy, respectively. The I-FVI exceeded the cut-off value of 0.83 except for five items from the attitude domain and four from the practice domains. Thus, seven of these items were revised to increase the clarity of items, while another two were deleted due to low I-FVI scores. Otherwise, the S-FVI/Ave for every domain exceeded the cut-off point of 0.9, which is considered acceptable. Thus, 50-item MUAPHQ C-19 (Version 3.0) was generated following the content and face validity analysis.

**Conclusions:**

The questionnaire development, content validity, and face validity process are lengthy and iterative. The assessment of the instruments’ items by the content experts and the respondents is essential to guarantee the instrument’s validity. Our content and face validity study has finalised the MUAPHQ C-19 version that is ready for the next phase of questionnaire validation, using Exploratory and Confirmatory Factor Analysis.

## Introduction

The coronavirus disease 2019 (COVID-19) is a global public health infection that terrifies the physical, mental, and social health of well-being [1–6]. Although many preventive and control measures have been implemented, including the COVID-19 vaccination programme, adherence to the new-normal post-COVID-19 is still the best preventative measure to be practised at the population level. The presence of an infodemic and fake news related to COVID-19 has led to a lot of misinformation about the virus [7]. This affects the people’s knowledge or understanding on COVID-19 [8] and subsequently, to their attitudes and practice [9–11].

It is essential to assess the community’s knowledge, attitude, practice (KAP), and health literacy on COVID-19 from time to time and analyse its associated factors to assist public health authorities in planning and implementing the appropriate preventive and control measures accordingly. In addition to the fluctuation trend of COVID-19, the announcement of COVID-19 endemicity in Malaysia on 1st April 2020 has shown how substantial the public compliance towards standard operational practices (SOPs) of the new normal is in ensuring they are protected from infections as well as its complication [1, 12].

KAP assessment is important in the evaluation for the prevention and control work [13, 14]. However, at the time of this study, most of the existing assessments of KAP on COVID-19 were developed by foreign country researchers [1]. None originated from Malaysia as the previous COVID-19 KAP study conducted in Malaysia has adapted the tool developed by Zhong B.L. et al. [15] from China. Furthermore, the variation on the study populations, questionnaire items, constructs, and type of measuring scales, making comparing the populations from different states and countries difficult or impossible when adapted and used locally. Besides, no other KAP questionnaire has incorporated the domain of health literacy about COVID-19 in the tool, especially in Malaysia. From the literature, the unspecified validation process reported from the published articles related to the assessment of KAP on COVID-19 is another lacking aspect that needs to be looked into [16–20]. Most of the KAP assessment tools on COVID-19 developed before were suited to the pandemic situation of COVID-19.

Developing a valid research tool is time-consuming. Nevertheless, a valid tool is needed to ensure better quality data with high comparability and credibility of the data [21]. Despite the construct validity, criterion validity and reliability are essential and frequently reported; content and face validity of a new tool is also crucial and are welcome to be reported [22]. This paper aims to describe the development of the Malay language of understanding, attitude, practice, and health literacy questionnaire on COVID-19 (MUAPHQ C-19) and subsequently to determine the content and face validity index of the MUAPHQ C-19.

## Methods

### Study design

**Fig. 1:**
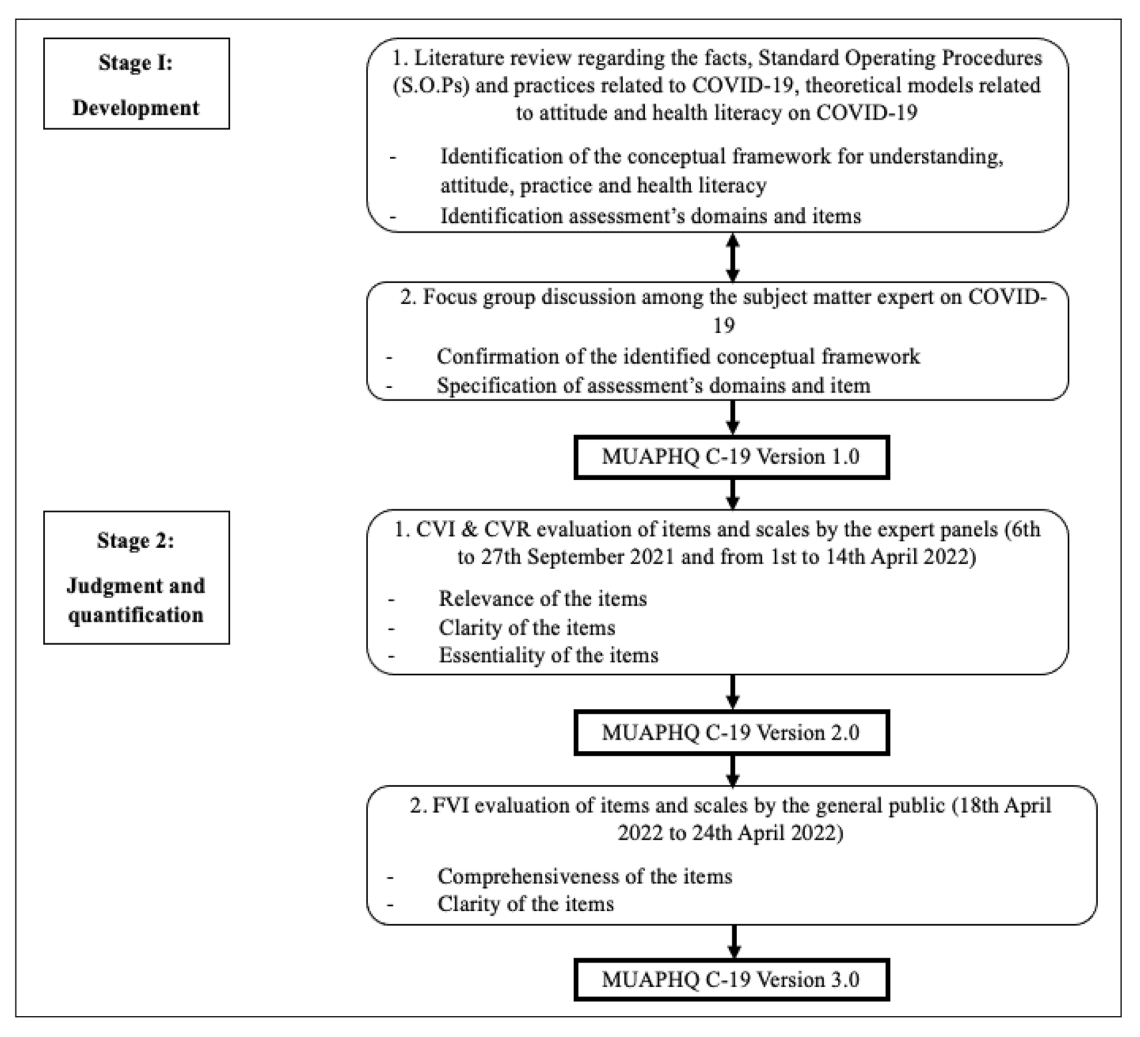
Stages of the development and validity of the MUAPHQ C-19

A cross-sectional study was conducted at the outpatient department of Universiti Teknologi MARA (UiTM) Specialist Centre (PPUiTM) Sungai Buloh, Selangor, Malaysia. A judgmental sampling method was employed to invite the expert panels for content validity based on their expertise and credibility. Purposive sampling was employed to select respondents for face validity. Figure 1 depicts the two-stage approach implemented for the development and validity of the MUAPHQ-C19, - stage I results in the generation of the instrument’s items (development), and stage II evaluates the performance of the instrument’s items (judgement and quantification) [23].

### Stage I. Development of the MUAPHQ C-19 questionnaire

The development of the MUAPHQ C-19 was conducted based on the comprehensive literature review and focus group discussion among the subject matter expert on COVID-19. Two questionnaires from the published articles by Azlan A.A. et al. [7] and Zhong B.L. et al. [15] were highlighted and modified to contribute to the item generation. Based on the literature review results and the focus group discussion among the subject matter expert on COVID-19, the conceptual framework and the first draft of the instrument (MUAPHQ C-19 Version 1.0) containing four domains (understanding, attitude, practice, and health literacy) was created.

### Stage II. Judgement and quantification of the MUAPHQ C-19 questionnaire

Two evaluations were included in this stage. Firstly, the assessment by the expert panels focusing on the content validity (relevancy and clarity) of the items in the MUAPHQ C-19 questionnaire and followed by the assessment of the face validity (clarity and comprehensiveness) of the items by the Malaysian public above 18 years old.

#### Expert panel

A group of six expert panels from the related field were invited to review the MUAPHQ C-19 (Version 1.0), they were:

i. two epidemiologists/ public health medicine physicians,
ii. one infectious disease microbiologist,
iii. one expert on health promotion and the Health Believe Model study,
iv. one clinical psychologist who is also a certified Malay language expert; and.
v. one statistician and questionnaire development expert.

#### Measurement of the content validity

The six expert panels evaluated the MUAPHQ C-19 (Version 1.0) based on four attributes, i.e., relevance, clarity, simplicity, and ambiguity. The questionnaire was reviewed and rated accordingly. All the expert panels attained a consensus on the content of the questionnaire. Each questionnaire domain was evaluated using the content validity index (CVI) [24].

Every item was rated on a four-interval scale (1 = not relevant; 2 needs some revision; 3 = needs minor revision, and 4 = very relevant) [23, 25]. The ratings given by the panel experts were used to calculate the CVI. Ratings of 1 and 2 represent invalid content or not a relevant item, while a rating of 3 and 4 represents relevant content. The experts could also provide comment on every item. A CVI was calculated in three indexes which are the item-level (I-CVI), scale-level (S-CVI), and content validity ratio (CVR) for each item’s relevancy [23, 25].

The I-CVI was calculated by the number of experts providing a score of 3 or 4 divided by the total number of experts [23]. Values can range from 0 to 1. The calculated I-CVI of 0.79 or more indicates the items are relevant, and 0.70 to 0.79 indicates the item needs revisions. In contrast, less than 0.70 suggests that items need to be eliminated. With more than five experts, the acceptable value for I-CVI is 0.78 [23, 26].

The S-CVI was calculated in two methods [26]:

i. the proportion of the items on one scale rated 3 or 4 by all the experts (universal agreement (UA) by experts) divided by the total number of items = S-CVI/UA and.
ii. the sum the I-CVI scores of all items divided by the total number of items (average of the I-CVI scores for all items across all experts) = S-CVI/Ave.

The S-CVI/UA is sensitive to the number of experts: the more experts are involved, the greater the possibility of obtaining a low S-CVI. This is because the universal agreement (UA) score is given as 1 when the item achieves all experts in agreement. Otherwise, the UA score is given as 0. The S-CVI/Ave is more liberal and is preferred by Polit D.F. et al. [26]. The S-CVI value of 0.8 or higher is considered acceptable [26]. Based on the values achieved by the items for I-CVI and S-CVI, the MUAPHQ C-19 was fine-tuned by the research team.

The CVR was calculated to measure an item’s essentiality, ranging from − 1 to + 1. The experts rated every item on a four-interval scale (1 = not necessary, 2 = useful but not essential, 3 = may be essential, and 4 = definitely essential). Ratings 1 and 2 represent “not essential”, while 3 and 4 represent “essential” content. The CVR is calculated by dividing the subtraction product of the number of panellists indicating an item as “essential” and half the number of panellists with half the number of panellists (CVR=(ne-N/2)/(N/2)) [27, 28]. The higher the score, the greater the agreement among the expert panels on the items’ necessity. The minimum CVR value for the six-panel experts involved is 0.99 [27]. Nevertheless, it is acceptable for a CVR value of at least 0.78; otherwise, the individual item should be revised or deleted [28].

#### Measurement of the face validity

The face validity was quantitatively assessed by the clarity and comprehensibility of all items in each domain through the respondents’ rating. Respondents rated the clarity and comprehensibility for all items in each domain according to a four-interval scale (1 = Not clear and understandable; 2 = Somewhat clear and understandable; 3 = clear and understandable, and 4 = Very clear and understandable). Ratings 1 and 2 represent invalid or irrelevant content, while ratings 3 and 4 represent valid or relevant content based on its clarity and comprehension [29].

There are two forms of face validity index (FVI) i.e., item-level FVI (I-FVI) and scale-level FVI (S-FVI) [29]. The I-FVI was calculated by dividing the number of items rated as 3 or 4 (agreed item) to the number of raters. S-FVI/Ave was calculated by averaging the I-FVI scores for all items across all raters. Values can range from 0 to 1. The calculated FVI of at least 0.83 was taken as the acceptable FVI value [29–31].

### Statistical analysis

Microsoft Excel was used for data entry and tabulation of CVI, CVR, and FVI.

## Results

### Stage I: development

Four domains, namely the understanding, attitude, practice, and health literacy towards COVID-19, were identified in the MUAPHQ C-19 conceptual framework. The summary of the MUAPHQ C-19 (Version 1.0) with 54 items is shown in Table 1. The understanding domain comprised 12 items. The literature review found that most of the studies, including Azlan A.A. et al. [7] and Zhong B.L. et al. [15], have included items that covered clinical presentation, transmission routes and prevention and control in their knowledge domain. Besides, the options for answers were categorical, i.e., True, False or Not sure. Our study has included items that not only cover the three themes mentioned above but also have added items related to the source of information, the infective agent, risk factors and complications of COVID-19. An interval from 1 (lowest understanding) to 10 (highest understanding) was used as the scale of measurement for the domain to give more freedom to the respondent in rating their understanding towards COVID-19 following each statement given compared to a specific yes or no answer.

Health Belief Model (HBM) theory was used as a guide in developing items of the attitude domain in this study. This is supported by the literature review finding that a study from Iran [32] and China [33] has adopted the same theory in the development of items in the attitude domain of their tool. Thus, sixteen items were identified in this domain, with four subdomains raised following the HBM theory [34]: Perceived Susceptibility, Perceived Severity, Perceived Benefit, and Perceived Barriers. Besides, instead of using an ordinal scale for the answer’s option like many other studies, an interval scale from 1 (strongly disagree) to 10 (strongly agree) was used in this study to measure the respondent’s agreement towards the statements on attitudes towards COVID-19.

The practice domain comprised 13 items. Initially, there were four subdomains raised from the practice domain, namely the 3 C’s (crowded place, confined space, and close contacts), the 3 W’s (wash hands, wear a mask, and warn the danger), the M.E.N. (touching mouth, eyes, and nose) and the WOMEN (wash hand, obey Standard Operating Procedure (S.O.P.), mask-up, exercise, and eat well, no unnecessary travelling). However, the subdomains were further divided into two subdomains, i.e., the practice to be done or Do’s (7 items) and the practice to be avoided or Don’ts (6 items). These items cover the preventive practices needed to be compliant during the COVID-19 pandemic. Instead of only adopting the three items from the study done by Azlan A.A. et al. [7] and Zhong B.L. et al. [15] (avoiding crowded places, wearing face masks when leaving home and practising proper hand hygiene), some items have been added to this study following the updated Standard Operating Procedure (S.O.P.) announced by the Malaysia authorities. This included practices on going to confined spaces places, the physical distancing of at least 1 m, usage of public transport, practices of shaking hands, touching mouth, eyes and nose, practices of showering after coming back from public areas, practices on updating COVID-19 status in “MySejahtera” applications, as well as practices related to test, report, isolate when having symptoms, or tested positive COVID-19. The interval scale from 1 (very rare) to 10 (very frequent) was used to measure the practices towards COVID-19 in the practice domain.

The health literacy domain, including 13 items, was developed based on the Health Literacy Survey (HLS-EU-Q47) framework [35] and a study conducted in Germany [36]. Despite no specific procedure in selecting items for the domain following the two mentioned studies, the HLS-EU-Q47 framework and the study by Okan et al. [36] have guided the researcher in developing the item following the four essential aspects of health literacy, namely the access, understand, appraise and apply. The four aspects of health literacy were adapted as the subdomains for the health literacy domain in this study. Besides, based on the review of the researchers, the majority of the items in the questionnaire used by the Okan et al. [36] study were quite general, especially when they mentioned protective behaviours or measures. However, in this study, the research team decided to develop a more focused item that can measure the literacy of the public in terms of “COVID-19 screening”, “getting treatment for COVID-19”, “self-protective measures”, “vaccination”, and “compliance to S.O.P”. The research team also decided to maintain the respondents’ freedom in rating the statements in the questionnaire. Thus, the interval scale from 1 (strongly disagree) to 10 (strongly agree) was used for the health literacy domain to measure the agreement with the statement.

**Table 1:** The domain, sub-domain and number of items of MUAPHQ C-19 (Version 1.0)

Domain	Sub-domain	No. of item	Total items	
Understanding	Source of information about COVID-19	1	12	
	Causative agent	2		
	Route of transmission	2		
	Symptom	1		
	Risk factor	1		
	Complication	1		
	Preventive measures for COVID-19	4		
Attitude	Perceived susceptibility	4	16	
	Perceived severity	5		
	Perceived benefits	2		
	Perceived barrier	5		
Practice	Do’s	7	13	
	Don’ts	6		
Health Literacy	Access health information	3	13	
	Understand health information	4		
	Appraise health information	4		
	Apply health information	2		
**Total no. of items**				**54**

### Stage II. Judgement and quantification of the MUAPHQ C-19 questionnaire

#### Content validity analysis

Based on the expert panel’s judgement, the I-CVI, UA and CVR for all four domains in MUAPHQ C-19 were calculated and shown in Table 2 (understanding domain), Table 3 (attitude domain), Table 4 (practice domain) and Table 5 (health literacy domain).

**Table 2:** The summary items (I-CVI, UA and CVR) which were retained, revised, and deleted for the understanding domain of MUAPHQ-C19 (Version 1.0)

	Item	No. of panel agree on the relevancy of item	I-CVI	UA	CVR	Comments	Decision	New item after revision	
U1	*I understand almost all the information shared regarding COVID-19 infection.*	6	1	1	1	-	Retained	-	
U2	*I understand that the COVID-19 infection is very dangerous to humans.*	6	1	1	1	-	Retained	-	
U3	*I understand that COVID-19 can reinfect to the same individual.*	6	1	1	1	-	Retained	-	
U4	*I understand how the transmission of the COVID-19 infection can occur between humans.*	5	0.8	0	0.7	Only 1/6 panel commented that the item needs to be more relevant, clear, complex, ambiguous and not essential. CVR is not met. However, no comments or suggestions were received from the other panels. Thus, the item is retained first at this stage. To be revised based on the face validity result.	Retained	-	
U5	*I understand the environmental conditions that can lead to the spread of the COVID-19 infection.*	6	1	1	1	4/6 panels suggested to amend the statement by adding the example of *“environmental conditions”* although they rated as 3 & 4. Since the essentiality of the item is good, the item is remained and revised	Revised	*I understand that environmental conditions such as confined with poor ventilation systems can cause the spread of the COVID-19 infection.*	
U6	*I understand all the symptoms related to the COVID-19 infection.*	6	1	1	1	-	Retained	-	
U7	*I understand that there are certain group of people that are categorized as high-risk groups of getting COVID-19 infection.*	6	1	1	1	-	Retained	-	
U8	*I understand the complications that can occur due to COVID-19 infection.*	6	1	1	1	-	Retained	-	
U9	*I understand that COVID-19 infection is preventable.*	6	1	1	1	-	Retained	-	
U10	*I understand that public health prevention practices such as wearing face masks, physical distancing and hand hygiene are the best ways to prevent the spread of COVID-19 infection.*	6	1	1	1	-	Retained	-	
U11	*I understand that COVID-19 vaccination can reduce the seriousness of COVID-19 infection.*	6	1	1	1	-	Retained	-	
U12	*I understand that the treatment of COVID-19 infection is given according to the category of infection*	6	1	1	1	-	Retained	-	
**S-CVI/UA**			0.92						
** S-CVI/Ave**			0.99						

Ave: Average; CVR: Content Validity Ratio; I-CVI: Item-level Content Validity Index; S-CVI: Scale-level Content Validity Index; UA: Universal Agreement

**Table 3:** The summary items (I-CVI, UA and CVR) which were retained, revised, and deleted for the attitude domain of MUAPHQ-C19 (Version 1.0)

	Item	No. of panel agree on the relevancy of item	I-CVI	UA	CVR	Comments	Decision	New item after revision
A1	*I perceive that the risk of me getting infected with COVID-19 is high.*	6	1	1	1	-	Retained	-
A2	*I perceive that COVID-19 infection is easy to infect from one individual to another.*	6	1	1	1	-	Retained	-
A3	*I perceive that I am easily infected with COVID-19.*	6	1	1	0.7	3/6 Panels commented that this item is similar to item A1. 1/6 panels commented that the item is not essential. CVR is not met. Thus, the item is deleted.	Deleted	-
A4	*I perceive that the risk of contracting with COVID-19 is high when I attend gatherings.*	5	0.8	0	1	1/6 panel suggested that the item should not be specific to “*gatherings*” only and should be generalised to a crowded place. Thus, the item is retained and revised.	Revised	*I perceive that the risk of contracting with COVID-19 is high when I go to crowded places.*
A5	*I perceive that the probability of me getting a critical level of COVID-19 infection is low.*	6	1	1	1	-	Retained	-
A6	*I perceive that COVID-19 infection poses a threat to public health.*	6	1	1	1	-	Retained	-
A7	*I perceive that COVID-19 infection will continue to be in the community at a low level.*	6	1	1	1	-	Retained	-
A8	*I perceive that COVID-19 infection can be controlled successfully in Malaysia*	6	1	1	1	-	Retained	-
A9	*I perceive that the complications of COVID-19 infection can involve all the body’s internal organs.*	6	1	1	1	-	Retained	-
A10	*I perceive that the COVID-19 infection can be prevented by wearing a face mask.*	6	1	1	1	-	Retained	-
A11	*I perceive that the COVID-19 infection can be prevented through physical distancing.*	6	1	1	1	-	Retained	-
A12	*I perceive that COVID-19 patients will be discriminated by the community.*	6	1	1	1	1/6 panel suggested a better arrangement of the item statement. Thus, the item is retained and revised.	Revised	*I perceive that COVID-19 patients will face discrimination in the community.*
A13	*I perceive that information about COVID-19 shared by the government is difficult to understand.*	6	1	1	1	-	Retained	-
A14	*I perceive that the difficulty in obtaining a stock of face masks is the main obstacle in the SOP compliance.*	5	0.8	0	0.7	1/6 panel commented that the item is irrelevant, unclear, simple, ambiguous and not essential as the face mask is now easily available in the market. The panel suggested the item be deleted. However, justification is made as the issue of accessibility of the face mask may remain a problem in some outskirt areas. Given that the other panels commented that the item is relevant, clear, simple, not ambiguous and essential, thus the item has remained, although the CVR is not met. One suggestion to revise the item is considered. Hence, the item is retained and revised.	Revised	*I perceive that the difficulty in obtaining face mask is the main obstacle in the SOP compliance.*
A15	*I perceive that it is difficult to get the latest information about the new norm instructions after been updated.*	5	0.8	0	0.7	1/6 panel commented that the item is irrelevant, unclear, simple and not essential, and 2/6 panel commented that the item is ambiguous. This refers to Malaysia going for endemicity now; thus, the panel suggested eliminating the item. However, justification is made as updates on COVID-19 are still available through various channels, even though we are entering the endemic phase of COVID-19. Given the guidelines and instructions of the new norms still keep changing; thus, the item is retained, although the CVR still needs to be met. One suggestion to revise the item is considered. Hence, the item is maintained and revised.	Revised	*I perceive that there is difficulty in getting updated information about the new norms after been updated by the authorities.*
A16	*I perceive the negative influence from the community makes it difficult for me to comply with the SOP.*	6	1	1	1	1/6 panel commented that the item needs to be clarified and ambiguous. The item has been revised	Revised	*I think the negative influence from the community influences me to comply with the SOP.*
**S-CVI/UA**			0.81					
** S-CVI/Ave**			0.97					

Ave: Average; CVR: Content Validity Ratio; I-CVI: Item-level Content Validity Index; S-CVI: Scale-level Content Validity Index; UA: Universal Agreement

**Table 4:** The summary items (I-CVI, UA and CVR) which were retained, revised, and deleted for the practice domain of MUAPHQ-C19 (Version 1.0)

	Item	No. of panel agree on the relevancy of item	I-CVI	UA	CVR	Comments	Decision	New item after revision
P1	*I went to a crowded place.*	6	1	1	1	-	Retained	-
P2	*I went to a confined and closed place.*	6	1	1	1	-	Retained	-
P3	*I talked to other people with a distance of more than a meter.*	6	1	1	1	-	Retained	-
P4	*I shake hands when see other people.*	6	1	1	1	2/6 panels suggested for substitution of the word *“see*”. Thus, the item is retained and revised.	Revised	*I shake hands when I meet other people.*
P5	*I use public transport.*	6	1	1	1	-	Retained	-
P6	*I often touch my eyes, nose or mouth.*	6	1	1	1	-	Retained	-
P7	*I always wear a face mask when I leave the house.*	6	1	1	1	-	Retained	-
P8	*I wash my hands regularly either with soap and water or using hand sanitizer*	6	1	1	1	-	Retained	-
P9	*I often take a shower after came back from a public place.*	6	1	1	1	-	Retained	-
P10	*I regularly update the status on my MySejahtera application.*	6	1	1	1	-	Retained	-
P11	*I will do a COVID-19 self-test if I have symptoms of COVID-19 infection.*	6	1	1	1	-	Retained	-
P12	*I will report the result of my COVID-19 screening test on the MySejahtera application*	6	1	1	1	-	Retained	-
P13	*I will self-isolate myself if I found positive for COVID-19 infection.*	6	1	1	1	-	Retained	-
**S-CVI/UA**			1.00					
** S-CVI/Ave**			1.00					

Ave: Average; CVR: Content Validity Ratio; I-CVI: Item-level Content Validity Index; S-CVI: Scale-level Content Validity Index; UA: Universal Agreement

**Table 5:** The summary items (I-CVI, UA and CVR) which were retained, revised, and deleted for the practice domain of MUAPHQ-C19 (Version 1.0)

	Item	No. of panel agree on the relevancy of item	I-CVI	UA	CVR	Comments	Decision	New item after revision
HL1	*I know how to get a verified information related to the prevention of COVID-19.*	6	1	1	1	-	Retained	-
HL2	*I know how to verify information about COVID-19 infection.*	6	1	1	1	1/6 panel suggested to revise the item to improve the clarity of the statement. The item is retained and revised.	Revised	*I know how to verify the truth of an information related to COVID-19 infection.*
HL3	*I know how to utilise the daily information shared by the Malaysian Ministry of Health (MOH) and the National Security Council (NSC).*	4	0.7	0	0.3	2/6 panels commented that the item is not relevant, not clear, complex, ambiguous and not essential. CVR is not met. Thus, the item is deleted.	Deleted	-
HL4	*I understand the appropriate time for me to do a screening test for COVID-19.*	6	1	1	1	1/6 panel suggested a better arrangement of the item statement. Thus, the item is retained and revised.	Revised	*I understand the right time for me to do the COVID-19 screening test.*
HL5	*I understand the importance of quarantine in preventing the spread of COVID-19 infection.*	6	1	1	1	-	Retained	-
HL6	*I understand the importance of close contact tracing.*	6	1	1	0.7	1/6 panel commented that the item is not essential. CVR is not met. However, no comments or suggestions were received from the other panels. Thus, the item is retained first at this stage. To be revised based on the face validity result.	Retained	-
HL7	*I understand of should be done if I have the symptoms of COVID-19 infection.*	6	1	1	1	-	Retained	-
HL8	*I know when I need to get treatment for COVID-19 infection.*	6	1	1	1	-	Retained	-
HL9	*I know when I need to practice self-prevention.*	6	1	1	1	-	Retained	-
HL10	*I know how to report if I find someone not complying with the “Standard Operating Procedures” (SOP).*	6	1	1	1	1/6 panel suggested a better arrangement of the item statement. Thus, the item is retained and revised	Revised	*I know how to make a report if I find someone not complying with the “Standard Operating Procedure” (SOP).*
HL11	*I understand about the effectiveness of the vaccine in protecting myself from severe COVID-19 infection or death.*	6	1	1	1	-	Retained	-
HL12	*I will make sure that I have gotten the COVID-19 vaccine to protect myself from the infection.*	6	1	1	1	1/6 panel suggested a better arrangement of the item statement. Thus, the item is retained and revised.	Revised	*I will make sure to get vaccinated to protect myself from severe infections.*
HL13	*I compliant to the “Standard Operating Procedures” (SOP) recommended by the government.*	6	1	1	1	-	Retained	-
**S-CVI/UA**			0.92					
**S-CVI/Ave**			0.97					

Ave: Average; CVR: Content Validity Ratio; I-CVI: Item-level Content Validity Index; S-CVI: Scale-level Content Validity Index; UA: Universal Agreement

Results showed that the I-CVI values of all 54 items in the MUAPHQ C-19 (Version 1.0) exceed the cut-off value of 0.78, except for one item from the health literacy domain with the I-CVI of 0.7 (HL3). The S-CVI values also exceeded the cut-off point of 0.8, i.e., S-CVI/UA (Overall) was 0.91, and S-CVI /Ave (Overall) was 0.98. The S-CVI/UA and S-CVI/Ave per domain were also calculated and showed acceptable values (> 0.8).

The CVR calculations showed that 6 out of 54 proposed items in MUAPHQ C-19 did not achieve the cut-off value of 0.99 (based on six expert panels), which were items U4, A3, A14, A15, HL3, and HL6. The value ranges from 0.3 to 0.7. Hence, two items were deleted (A3 and HL3), and four others were revised. On the other hand, 9 out of 48 items that have achieved the CVR cut-off value were revised too based on the recommendations and consensus of the expert panels. The summary of the items for all domains is shown in Table 6.

**Table 6:** The summary of the items for all domains in MUAPHQ C-19 (Version 2.0) following the content validity analysis

Domains	No of items	No of the items revised before being retained	No. of items deleted	No. of items retained
Understanding	12	1	-	12
Attitude	16	5	1	15
Practice	13	1	-	13
Health Literacy	13	4	1	12
**Total**	**54**	**11**	**2**	**52**

#### Face validity analysis

After the content validity analysis, the MUAPHQ C-19 (Version 2.0) containing 52 items was further analysed for the face validity index. There were ten respondents who participated in the face validity analysis. A total of 60% were female, ranging from 18 to 71 years old. Fifty per cent of the participants were Malay, followed by three Chinese and two Indian. The majority, 70 per cent, of the participants, had the highest education level at the secondary level while the remaining up until tertiary level (degree level).

The I-FVI exceeded the cut-off value of 0.83 for all 52 items except for five items from the attitude domain and four from the practice domain (ranging from 0.6 to 0.8). Thus, seven of these items were revised to increase the clarity of the items based on the verbal feedback from the participants (items A5, A12, A13, A15, A16, P1 and P2), while another two items were deleted due to the low I-FVI scores and the suitability of the item to the management of COVID-19 in endemic phase in Malaysia (items P4 and P5). Regarding the S-FVI/Ave, all values for all domains exceeded the cut-off point of 0.9, which is considered acceptable. The summary of the items for all domains in MUAPHQ C-19 following the face validity analysis is shown in Table 7.

**Table 7:** The summary of the items for all domains in the MUAPHQ C-19 (Version 3.0) following the face validity analysis

Domains	No of items	No of the items revised before being retained	No. of items deleted	No. of items retained
Understanding	12	-	-	12
Attitude	15	5	-	15
Practice	13	2	2	11
Health Literacy	12	-	-	12
**Total**	**52**	**7**	**2**	**50**

## Discussion

This study aimed to assess the content and face validity index of the development of the understanding, attitude, practice and health literacy questionnaire on COVID-19 (MUAPHQ C-19) in Malay language. An approach suggested by Lynn M.R. [23] that has been widely used mainly in health care and nursing research has been employed in this study. Content validity is an important quality indicator of an instrument’s validity. The researchers developed the MUAPHQ C-19 (Version 1.0) containing a total of 54 items in four domains at the first stage, subsequently 52 items have been retained after conducting the content validity analysis, and finally retained 50 items post-face validity analysis.

Six-panel experts have been recruited to facilitate the content validation process, modifications and improvement of the instrument which was in the range recommended by Lynn M.R. [23]. The competency of the panel experts chosen is critical. They should be a person who represents the content of interest, familiar with the methodology of assessment, and so much use if they are from the stakeholders from additional application fields to the content of interest [37]. This study focused on panel experts with a strong background in public health and infectious disease. Two clinical epidemiologists and one infectious disease microbiologist have been recruited who were well-versed with COVID-19. In addition, an expert in conducting health promotion study as well as known with Health Belief Model study has been recruited, which is helpful to give advises on items in the instrument, particularly for the attitude and practice domain. A Malay language certified expert was also recruited, who played a critical role in ensuring the clarity and suitability of the wording and phrases in the Malay language in the instrument. Besides, a statistician who is also a questionnaire development expert was also recruited as one of the panel experts, which was helpful to ensure the development and validation process is on track and right. However, it was assumed that a small risk of bias as the panel expert’s feedback is subjective, and bias may exist among the experts [24]. The consensus among the panel experts is beneficial to come to a meeting point where all the feedback is considered and to finalise the interpretation and modification.

The use of CVI indices has been criticised because the calculation of the agreement indices and the significance of the number of experts in the rating can lead to the risk of error, thus, it was recommended supplementing the CVI indices with a multi-rater kappa coefficient. However, these agreement indices are only part of the content validity assessment and should not be the only reason for the rejection or modification of items [38]. Feedback and comments from the expert panels also be a form of judgement to supplement the content validity indices [38]. Present study employed the CVI results supplemented by consensus from the panel expert’s comments in modifying the MUAPHQ C-19 (Version 1.0) similar to the conduct of other studies [39, 40].

Following the response process represented by the FVI, all four domains scored a high level of face validity in terms of clarity and comprehensibility. This indicates a good response process. Despite the good scores of S-FVI/Ave for all items above the cut-off value of 0.83, some items scored I-FVI lesser than 0.83 [30, 41]. Besides, the qualitative comments from the respondents were still considered. Furthermore, due to the continuous updates on the Standard Operation Procedure for COVID-19 in Malaysia (during the conduct of this methodological study) when the country enters different phases of pandemics, some changes have been made to the MUAPHQ C-19 to address those concerns [12, 42], which is through rewording and rephrasing the statements to facilitate the clarity and comprehensibility of the items.

More methodological studies should be conducted in the future as it clarifies the acceptance of the developed tool not only from panel expert’s perspective, but also from the user’s perspective, especially when combining the qualitative and quantitative information for determining the validity [22]. In addition, it allows comparing the measures with other KAP tools related to COVID-19, particularly for Malaysian public health usage. It is hoped that the final version of MUAPHQ C-19 can be used as one of the continuous reference tools for COVID-19 KAP among the general public and provides information for better planning of COVID-19 prevention and control policy.

## Strength and limitations

This is the first study on the development of the UAPH tool in Malay that elaborates on the content validity and face validity analysis in detail. However, some limitations were identified. In this study, we didn’t calculate the multi-rate kappa that may provide more valid agreement indices to the content validity of MUAPHQ C-19. However, the report of both item and scale level CVI, together with considering comments from the panel experts, would suffice to validate the content of MUAPHQ C-19. Plus, the findings were supported by the acceptable FVI value for all domains. The other limitation is that the MUAPHQ C-19 was meant to be developed in Malay. Thus, there was no translation process incorporated in this study, and it may limit the usage of MUAPHQ C-19 to only the population that understands Malay in the future.

## Conclusion

Generally, the questionnaire development and content validity process are lengthy and iterative. The content and face validity study has finalised the 50-item MUAPHQ C-19 version that is ready for the next phase of questionnaire validation, namely exploratory factor analysis and confirmatory factor analysis. The 50-item MUAPHQ C-19 revealed an appropriate level of content and face validity based on its I-CVI, S-SCI/Ave, CVR and S-FVI/Ave indices.

## Data Availability

The datasets used and analysed during the current study are not publicly available and only shared with the research team due to their confidentiality and need to be protected, as stated by the Malaysia Ministry of Health Medical Research Ethics Committee. However, the datasets are available from the corresponding author upon reasonable request.

## Abbreviations

Ave: Average
CVI: Content validity index
CVR: Content validity ratio
FVI: Face validity index
I-CVI: Item-level content validity index
I-FVI: Item-level face validity index
S-CVI/Ave: Scale-level content validity index/average
S-CVI/UA: Scale-level content validity index/universal agreement
S-FVI/Ave: Scale-level face validity index/average
